# CD19 Alterations Emerging after CD19-Directed Immunotherapy Cause Retention of the Misfolded Protein in the Endoplasmic Reticulum

**DOI:** 10.1128/MCB.00383-18

**Published:** 2018-10-15

**Authors:** Asen Bagashev, Elena Sotillo, Chih-Hang Anthony Tang, Kathryn L. Black, Jessica Perazzelli, Steven H. Seeholzer, Yair Argon, David M. Barrett, Stephan A. Grupp, Chih-Chi Andrew Hu, Andrei Thomas-Tikhonenko

**Affiliations:** a Division of Experimental Pathology, Children's Hospital of Philadelphia, Philadelphia, Pennsylvania, USA; b Division of Oncology, Children's Hospital of Philadelphia, Philadelphia, Pennsylvania, USA; c Protein and Proteomics Core Facility, Children's Hospital of Philadelphia, Philadelphia, Pennsylvania, USA; d Immunology, Microenvironment and Metastasis Program, The Wistar Institute, Philadelphia, Pennsylvania, USA; e Perelman School of Medicine at the University of Pennsylvania, Philadelphia, Pennsylvania, USA

**Keywords:** RNA splicing, immunotherapy, membrane proteins, membrane transport, protein folding

## Abstract

We previously described a mechanism of acquired resistance of B-cell acute lymphoblastic leukemia to CD19-directed chimeric antigen receptor T-cell (CART) immunotherapy. It was based on in-frame insertions in or skipping of CD19 exon 2. To distinguish between epitope loss and defects in surface localization, we used retroviral transduction and genome editing to generate cell lines expressing CD19 exon 2 variants (CD19ex2vs) bearing vesicular stomatitis virus G protein (VSVg) tags. These lines were negative by live-cell flow cytometry with an anti-VSVg antibody and resistant to killing by VSVg-directed antibody-drug conjugates (ADCs), suggestive of a defect in surface localization. Indeed, pulse-chase and α-mannosidase inhibitor assays showed that all CD19ex2vs acquired endoplasmic reticulum (ER)-specific high-mannose-type sugars but not complex-type glycans synthesized in the Golgi apparatus. When fused with green fluorescent protein (GFP), CD19ex2vs (including a mutant lacking the relevant disulfide bond) showed colocalization with ER markers, implying protein misfolding. Mass spectrometric profiling of CD19-interacting proteins demonstrated that CD19ex2vs fail to bind to the key tetraspanin CD81 and instead interact with ER-resident chaperones, such as calnexin, and ER transporters involved in antigen presentation. Thus, even the intact domains of CD19ex2vs cannot be easily targeted with ADCs or current CD19 CARTs but could serve as sources of peptides for major histocompatibility complex (MHC)-restricted presentation and T-cell receptor (TCR)-mediated killing.

## Abstract (precis)

We previously described a mechanism of acquired resistance of B-cell acute lymphoblastic leukemia to CD19-directed chimeric antigen receptor T-cell (CART) immunotherapy. It was based on in-frame insertions in or skipping of CD19 exon 2.

## INTRODUCTION

CD19-targeting immunotherapies, such as the bispecific T-cell engager (BiTE) blinatumomab, are effective at treating relapsed and refractory pediatric B-cell acute lymphoblastic leukemia (B-ALL) (1, 2). More recently, chimeric antigen receptor (CAR)-armed T cells targeting CD19 (CART-19) were developed and shown to yield 70% to 80% complete response rates in pediatric patients (3, 4). These gains culminated in the recent FDA approval of tisagenlecleucel and axicabtagene ciloleucel for patients with refractory/relapsed B-cell malignancies. However, on both therapies, 20% to 30% of patients do not respond or do not stay in remission for more than several weeks. Nearly half of the relapses following CART-19 occur due to the failure of infused T cells to engraft or propagate, in which case reinfusion remains a viable therapeutic option. The other half of relapsed leukemias lose CD19 antigen expression on the cell surface and thus are referred to as surface CD19 (sCD19) negative. Such patients could be treated with alternative CARs such as CART-22; however, the observed responses are typically not as durable as those seen with anti-CD19 therapies, with CD22-low/negative relapses emerging posttreatment (5). While strategies based on dual-antigen targeting are beginning to emerge (6), the apparent superiority of CD19 as a leukemia-associated antigen fuels interest in alternative CD19-directed therapeutics recognizing epitopes other than the commonly used FMC63 (7, 8).

While in some sCD19-negative relapses CD19 expression is extinguished at the level of dedifferentiation into myeloid lineages (9, 10), we recently described a posttranscriptional mechanism of acquired resistance to CART-19 compatible with the B-cell phenotype (11; reviewed in references 12 and 13). It is based on the selective loss of CD19 extracellular and transmembrane domains, primarily through skipping of exon 2, which encodes the first of the two constant region 2 (C2)-type Ig-like loops (14). Since then, preexisting alternative splicing variants have been observed in pediatric and adult patients with B-ALL (15). In principle, such variants could be targeted with alternative immunotherapies directed, for example at the second C2 loop, as long as surface expression of CD19 is maintained. Interestingly, another sCD19-negative relapse we analyzed, CHOP105R, harbored an in-frame 9-nucleotide insertion in exon 2, but sCD19 loss did not appear to involve alternative splicing (11). Here, we describe the nature of sCD19 loss resulting from mutations that do not affect alternative splicing.

Proteins destined for the cell surface enter the endoplasmic reticulum (ER) lumen guided by the signal peptide (SP) sequence (16, 17). Once in the ER, the protein undergoes cotranslational N-linked glycosylation, as well as folding and assembly catalyzed by disulfide formation (18–20). Next, these proteins are translocated to the Golgi apparatus, where mannose trimming and modifications by complex glycans occur before they are delivered to the cell surface. Mutations that alter proper protein folding and assembly could trigger ER retention of the misfolded protein (21, 22). With respect to CD19, potentially relevant amino acid sequences include a glycosylation site at Asp86 and the two amino acids involved in the disulfide bond formation (Cys38-Cys97).

We previously observed that the Δex2 isoform has an abnormal subcellular localization (11). Here, we investigated whether in-frame mutations in exon 2 were functionally similar to the skipping of exon 2 in that they, too, might have a defect in membrane localization resulting in CD19 negativity by flow cytometry. This scenario could not be directly tested by studying primary patient samples, since it would be impossible to distinguish between epitope loss and defects in cell surface localization. This necessitated extensive reverse engineering of B-ALL cell lines using clustered regularly interspaced short palindromic repeat (CRISPR)/Cas9 genome editing and incorporation of validated epitopes into the CD19 amino acid sequence.

## RESULTS

### Mutations in CD19 exon 2 result in poor epitope surface presentation.

To study CD19 exon 2 variants (CD19ex2vs) in B-ALL cells, we used CRISPR/Cas9 technology to knock out endogenous CD19 in B-ALL cell lines. We electroporated a Cas9-expressing plasmid and a mixture of several short guide RNAs (sgRNAs) into Nalm6 and 697 cells, isolated CD19-negative single-cell clones by flow cytometry, and confirmed gene targeting using Western blotting for full-length CD19. Sanger sequencing across exons 2 and 3 showed heterozygous insertions at the Cas9 cut sites, both of which lead to premature stop codons and translation termination (data not shown).

Next, to test if the immunotherapy-resistant CD19-105R (previously referred to as 105R2 [11]) and Δex2 variants can be tagged with alternative epitopes for flow cytometry, we generated multiple retroviral constructs expressing full-length CD19 (FL-CD19) and bearing hemagglutinin (HA), FLAG, vesicular stomatitis virus G protein (VSVg), and His tags. The tags were cloned into exon 1 or 3 to avoid interference with exon 2 mutations. The constructs were transduced into the Nalm6 CD19 knockout (KO) cells (Nalm6-ΔCD19) and were analyzed by immunoblotting and flow cytometry with FMC63- and tag-specific antibodies. Only VSVg and HA tags cloned immediately after the SP sequence in exon 1 resulted in robust detection by cytometry and immunoblotting, without interfering significantly with detection of the CART-19-targeted FMC63 epitope. The VSVg tag, however, yielded a stronger signal than the HA tag and was thus chosen for further experiments (data not shown).

**FIG 1:**
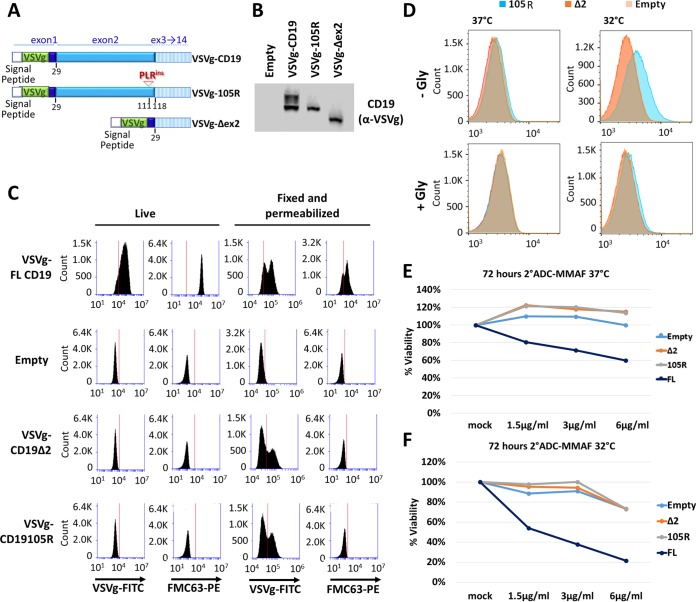
Mutations in CD19 exon 2 result in cell surface localization defects. (A) Schematic of VSVg-CD19 FL, VSVg-105R, and VSVg-Δex2 retroviral constructs. The VSVg tag was inserted in exon 1 right after the signal peptide sequence to prevent cleavage of the epitope. (B) Western blotting with anti-VSVg antibody in transduced Nalm6 cell lines. (C) Flow cytometry of live or fixed/permeabilized transduced cell lines stained with anti-VSVg–FITC or anti-CD19 (FMC63)–PE antibody. (D) Flow cytometry of live transduced Nalm6 cell lines grown at 37°C or 32°C for 72 h before staining with anti-VSVg–FITC for 1 h. The cells were then washed with PBS as a control or glycine to dissociate any surface-bound antibody. (E and F) Killing assays using anti-VSVg antibody and anti-mouse antibody–MMAF 2-ADC in transduced Nalm6 cell lines grown at 37°C (E) or 32°C (F) for 72 h.

We then generated, in the same manner, the VSVg-105R and VSVg-Δex2 CD19 variant constructs (Fig. 1A). We transduced those constructs in the Nalm6-ΔCD19 cells and detected robust protein expression by Western blotting using the VSVg antibody (Fig. 1B). Furthermore, when cells were fixed and permeabilized before staining for flow cytometry, both the VSVg-105R and VSVg-Δex2 CD19 variants showed intracellular reactivity with the VSVg antibody but not with the FMC63-phycoerythrin (PE) antibody, indicating complete loss of the cognate CART epitope (Fig. 1C, Fixed and permeabilized). However, staining of live cells using either anti-VSVg or anti-CD19 (FMC63) antibody detected sCD19 only in cells expressing the VSVg-FL construct (Fig. 1C, Live), while cells expressing VSVg-105R and VSVg-Δex2 CD19 mutants appeared sCD19 negative, indicating a defect in surface presentation.

Lowering the temperature of cell culture incubation can improve the surface expression of receptors that are absent from the plasma membrane due to mutations (23). To test whether CD19ex2vs proteins are temperature sensitive, we grew cells at 32°C and tested them for overall CD19 expression and surface presentation. After 3 days of culture at the permissive temperature, immunoblotting and reverse transcription-quantitative PCR (RT-qPCR) analysis showed increased expression of all CD19 variants (data not shown). However, flow cytometric analysis of live cells stained with the VSVg antibody showed weak positivity of VSVg-105R, while the VSVg-Δex2 variant remained negative (Fig. 1D, top). This positivity was abolished when stained cells were treated with glycine (Gly), which dissociates surface-bound/noninternalized antibodies (Fig. 1D, bottom).

To test whether increased surface presentation of VSVg-105R at 32°C was enough to trigger cell killing, we utilized the anti-mouse secondary antibody conjugated to monomethyl auristatin F (MMAF) (secondary-antibody–drug conjugate [2-ADC]). Incubation of VSVg–FL-CD19 cells grown at 37°C or 32°C with the anti-VSVg antibody and 2-ADC resulted in cell killing at both temperatures (Fig. 1E and F, dark-blue curves). In fact, we noted increased sensitivity to 2-ADC at 32°C, which could be due to higher expression levels of FL-CD19. However, neither VSVg-105R nor VSVg-Δex2 CD19 was susceptible to killing by 2-ADC at either temperature (Fig. 1E and F, orange and gray curves), consistent with their impaired surface localization.

### CD19ex2 variants exhibit altered glycosylation patterns.

**FIG 2:**
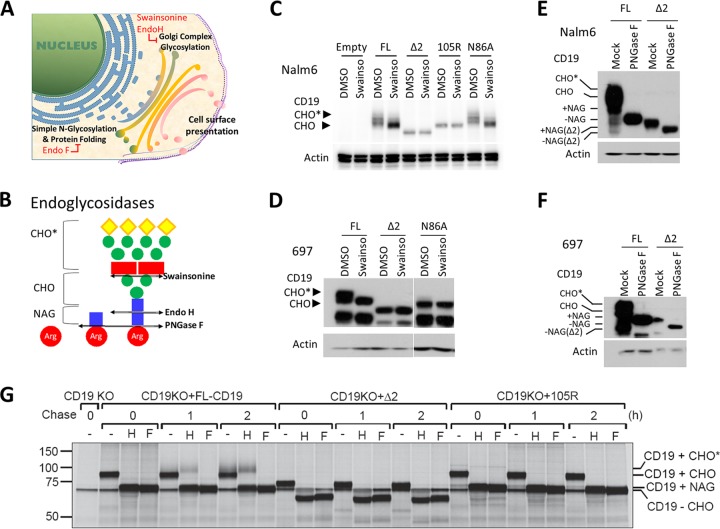
CD19ex2 variants exhibit altered glycosylation patterns. (A) Schematic showing cellular compartments where sequential steps of protein glycosylation and processing occur. (B) Schematic showing the intended targets of cleavage by endo H and endo F glycosidases, as well as the α-mannosidase inhibitor swainsonine. (C) Western blot with anti-CD19 or antiactin antibody of protein extracts from the transduced Nalm6 cell lines after treatment with DMSO or 10 μg/ml swainsonine for 24 h. (D) Western blot with anti-CD19 or antiactin antibody of lysates from the transduced 697 cell lines following treatment with DMSO or 10 μg/ml swainsonine for 24 h. (E) Western blot with anti-CD19 or antiactin antibody of protein lysates from the transduced Nalm6 cell lines following *in vitro* mock or PNGase F treatment. (F) Western blot with anti-CD19 or antiactin antibody of protein lysates from the transduced 697 cell lines following *in vitro* mock or PNGase F treatment. (G) Nalm6-ΔCD19 cells or cells transduced with CD19-FL or CD19ex2vs constructs were radiolabeled for 15 min, chased for 1 or 2 h, and immunoprecipitated using a monoclonal antibody against human CD19. Immunoprecipitates were treated with endo H (H) or PNGase F (F) before analysis on an SDS-PAGE gel. CHO, high-mannose-type glycans; CHO*, complex-type glycans; NAG, *N*-acetylglucosamine.

Since surface expression of CD19 depends on proper glycosylation and folding (14), we asked whether CD19 variants showed altered glycosylation. The nascent CD19 polypeptide is initially glycosylated in the ER, followed by addition of complex sugars in the Golgi apparatus (21, 22) (Fig. 2A). According to the Universal Protein Resource (UniProt) database (24), CD19 contains five asparagine (N) residues in the extracellular domain, one of which is located in exon 2 (N86). This is the only asparagine missing in the Δex2 CD19 variant, and in the 105R mutant, it is separated by only 25 amino acids from the Pro-Leu-Arg insertion and could in theory be masked by it. To investigate if glycosylation of the N86 residue is required for CD19 surface presentation, we generated the N86A-CD19, mutant which is expressed at levels similar to those of other CD19 variants. We then treated FL, N86A, Δex2, and 105R CD19-expressing cells for 24 h with swainsonine, an inhibitor of the complex glycosylation that occurs in the Golgi apparatus and is resistant to enzymes, such as endoglycosidase H (endo H) (25) (Fig. 2B).

Lysates of dimethyl sulfoxide (DMSO)- or swainsonine-treated cells were analyzed by Western blotting to determine whether, upon treatment, the CD19 band migrates faster, to reflect a decrease in Golgi apparatus-acquired glycosylation. As predicted, the full-length (FL) isoform was affected by swainsonine, indicating that it acquires complex Golgi apparatus glycosylation (Fig. 2C); actin served as a nonglycosylated loading control. Surprisingly, the N86A-CD19 mutant exhibited similar sensitivity to swainsonine, suggesting that glycosylation at asparagine residues other than N86 is sufficient for proper ER and Golgi apparatus processing (Fig. 2C). On the other hand, the electrophoretic mobilities of Δex2- and 105R-specific bands did not shift after treatment with swainsonine, indicating that these isoforms do not acquire complex sugar glycosylation and likely do not even reach the Golgi apparatus (Fig. 2C). We also tested the swainsonine sensitivities of these CD19 variants in CD19-KO 697 cells and observed identical results (Fig. 2D).

To understand the extent of defective glycosylation of CD19ex2vs, we performed *in vitro* treatment of Nalm6 and 697 cells expressing FL-CD19 or Δex2 CD19 with peptide-*N*-glycosidase F (PNGase F), which removes mannose and complex glycans (Fig. 2A and B). We observed gel shifts for both FL-CD19 and Δex2 CD19 following treatment in both Nalm6 and 697 cells (Fig. 2E and F), suggesting these isoforms do acquire high-mannose glycosylation in the ER. To further prove that Δex2 CD19 and 105R lack proper glycosylation processing, we performed pulse-chase experiments followed by deglycosylation of immunoprecipitated CD19 protein. While PNGase F is capable of removing mannose and complex glycans, endo H removes only mannose glycans that are not modified by complex-type glycans acquired in the Golgi apparatus (26, 27) (Fig. 2A and B). Interestingly, only FL-CD19 acquired endo H resistance, as evidenced by the higher-molecular-weight (higher-MW) band appearing at 1 and 2 h (Fig. 2G), suggesting that complex glycosylation of FL-CD19 occurs in the Golgi apparatus. In contrast, Δex2 CD19 and 105R mutants were still sensitive to endo H treatment after 2 h of chase, suggesting Golgi apparatus-acquired glycosylation does not occur. These data confirm that immunotherapy-resistant mutants do not reach the Golgi apparatus and are retained in the ER.

### Inhibition of CD19 glycosylation does not affect its subcellular localization.

**FIG 3:**
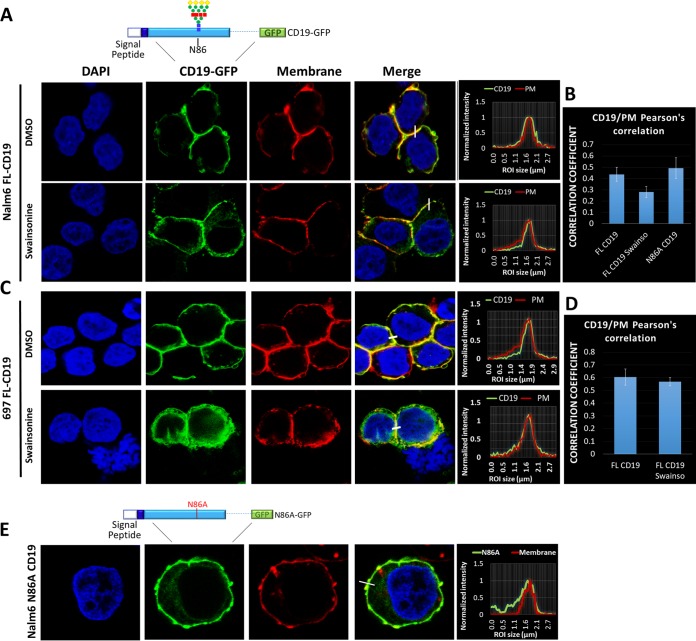
Inhibition of CD19 glycosylation does not affect its subcellular localization. (A) Immunofluorescence of Nalm6-ΔCD19 cells expressing FL-CD19–GFP (top schematic) after treatment with DMSO or swainsonine for 24 h. The plasma membrane was stained with wheat germ agglutinin-Alexa Fluor 647 (converted to red), and nuclei were stained with DAPI (blue). The histograms show colocalization of CD19-GFP (green) and plasma membrane (red) channels. (B) Pearson's correlation colocalization analyses for FL-CD19–GFP and plasma membrane. Three separate fields containing at least 100 cells were analyzed for each condition. The error bars indicate standard deviations. (C) Immunofluorescence of 697-ΔCD19 cells expressing CD19-FL-GFP after treatment with DMSO or swainsonine for 24 h. The plasma membrane was stained with wheat germ agglutinin-Alexa Fluor 647 (converted to red), and nuclei were stained with DAPI (blue). The histograms show colocalization of CD19-GFP (green) and plasma membrane (red) channels. (D) Pearson's correlation colocalization analyses of FL-CD19–GFP and plasma membrane. Three separate fields containing at least 100 cells were analyzed for each condition. (E) Immunofluorescence of Nalm6-ΔCD19 cells transduced with N86A-CD19–GFP fusion protein (top schematic). The plasma membrane was stained with wheat germ agglutinin-Alexa Fluor 647 (red).

To further understand the role that Golgi apparatus-acquired glycosylation plays in the surface presentation of CD19, we generated FL-CD19–green fluorescent protein (GFP) and N86A-CD19–GFP fusion proteins for confocal microscopy analysis and introduced them into the CD19-null Nalm6 cell line. The transduced cells were then cultured in the presence of swainsonine for 24 h. For colocalization studies, the cellular membranes were stained with wheat germ agglutinin. Visual examination of fluorescent signals (Fig. 3A) and Pearson's colocalization analysis of the confocal images (Fig. 3B) confirmed that inhibiting Golgi apparatus-acquired glycosylation did not significantly affect the localization of FL-CD19 on the plasma membrane. Identical results were obtained using the 697 cell line (Fig. 3C and D). Furthermore, as predicted by flow cytometric analysis, loss of Asp86 did not result in the loss of surface expression (Fig. 3E). Hence, altered glycosylation of exon 2-encoded amino acid residues cannot explain the immunotherapy-resistant phenotype of the Δex2 and 105R variants.

### CD19ex2 variants localize in the endoplasmic reticulum.

**FIG 4:**
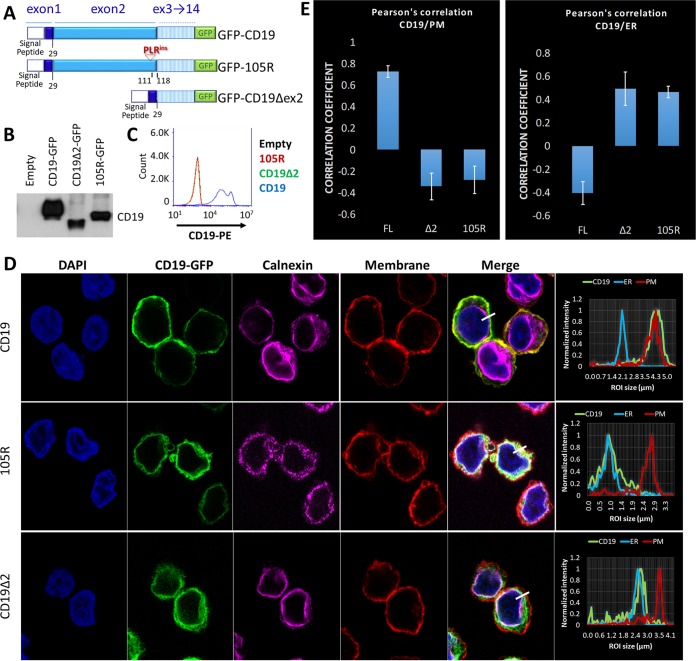
CD19ex2 variants localize in the endoplasmic reticulum. (A) Schematic of the CD19-FL, CD19Δex2, and CD19-105R–GFP fusion protein constructs expressed in Nalm6-ΔCD19 cells. (B) Western blot with anti-CD19 antibody of lysates from the indicated cell lines. (C) Live-cell flow cytometry for empty (black), CD19-FL (blue), CD19-Δex2 (green), and CD19-105R (red) using anti-CD19–PE antibody. (D) Immunofluorescence confocal microscopy of GFP construct-transduced cell lines (green). The plasma membrane was stained with wheat germ agglutinin-Alexa Fluor 647 (converted to red). The endoplasmic reticulum was stained with anticalnexin (Cell Signaling)/anti-rabbit antibody–Alexa Fluor 594 (converted to magenta). (Right) Histogram localization analysis showing overlaps of channels. (E) Pearson's correlation coefficient analyses showing strong colocalization of plasma membrane and CD19-FL and endoplasmic reticulum and CD19ex2vs isoforms. The error bars indicate standard deviations.

The results of the pulse-chase experiment suggested that immunotherapy-resistant CD19 variants acquire only high-mannose glycans and are retained in the ER. To determine whether immunotherapy-resistant CD19 mutants indeed reside in the ER, we generated Δex2-GFP and 105R-GFP constructs (Fig. 4A) and transduced them into the CD19-null Nalm6 cells. Immunoblotting with cytoplasmic antibody confirmed that all CD19-GFP variants were expressed at similar levels (Fig. 4B). In contrast, flow cytometric analysis using the FMC63-PE antibody showed that only FL-CD19–GFP-expressing cells exhibit surface expression of CD19 (Fig. 4C). For confocal microscopy, we stained the plasma membrane with wheat germ agglutinin (red channel) and an antibody against the ER-resident protein calnexin (magenta channel). FL-CD19–GFP (green channel) was almost exclusively expressed on the cell surface, as evidenced by the overlap of the green and red histograms (Fig. 4D, top). In contrast, the Δex2-GFP and 105R-GFP mutants strongly colocalized with calnexin (Fig. 4D, middle and bottom). This was confirmed by Pearson's colocalization analyses of GFP, calnexin, and cellular membrane fluorescent signals (Fig. 4E). These data further support our hypothesis that CD19ex2vs are retained in the ER and fail to translocate to the Golgi apparatus for processing and further trafficking to the cell surface.

### Disruption of the CD19 Cys38-Cys97 disulfide bond leads to loss of surface expression.

**FIG 5:**
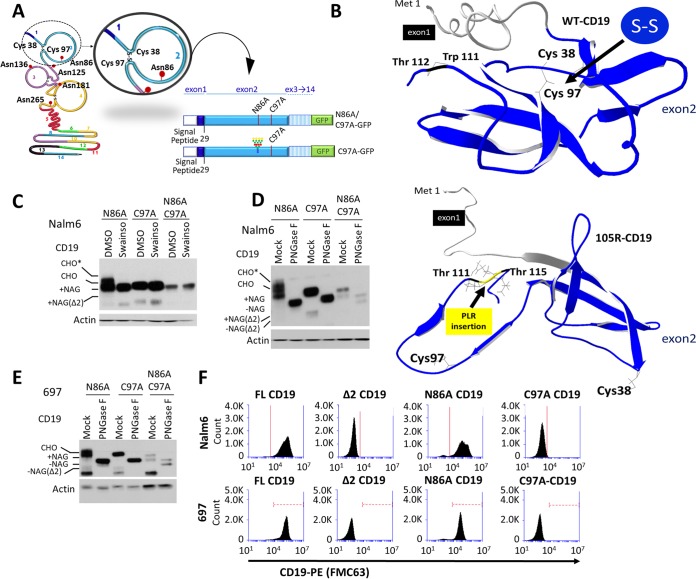
Disruption of the CD19 Cys38-Cys97 disulfide bond leads to loss of surface expression. (A) Schematic representation of the CD19 protein indicating the exon 2 residues involved in bisulfite bonds and loop formation and in glycosylation. Depictions of the C97A and N86A/C97A-CD19 protein constructs are also shown. (B) i-Tasser protein structure prediction of CD19-FL (top) and CD19-105R (bottom). For simplicity, only exons 1 and 2 are shown. (C) Western blot with anti-CD19 or antiactin antibody of protein extracts from the transduced Nalm6 cell lines following treatment with DMSO or 10 μg/ml swainsonine for 24 h. Various degrees of CD19 protein glycosylation are shown by gel shift of bands. CHO, high-mannose-type glycans; CHO*, complex-type glycans; NAG, *N*-acetylglucosamine. (D) Western blot with anti-CD19 or antiactin antibody of protein lysates from the transduced Nalm6 cell lines following *in vitro* mock or PNGase F treatment. (E) Western blot with anti-CD19 or antiactin antibody of protein lysates from the transduced 697 cell lines following *in vitro* mock or PNGase F treatment. (F) Live-cell flow cytometry using anti-CD19–PE antibody of transduced Nalm6 and 697 cell lines.

We next investigated whether abnormal protein folding was responsible for retention of CD19ex2vs in the ER. The extracellular region of CD19 has two Ig-like loops held together by Cys-Cys disulfide bonds (28). The second Ig-like loop involving Cys200 and Cys261 is encoded by exon 4 and thus was of limited relevance to our analysis. On the other hand, the first loop, which relies on Cys38-Cys97 interaction, is encoded entirely within exon 2 (Fig. 5A). Cys38 and Cys97 are missing in the Δex2 variant but are preserved in the 105R mutant, yet both CD19ex2vs are retained in the ER. To determine how the 105R mutation might affect loop formation, we analyzed the three-dimensional (3D) protein structure using i-Tasser prediction software (29). Predictably, it revealed that in FL-CD19, Cys38 and Cys97 are positioned in close proximity to each other (Fig. 5B, top). However, in 105R, the Pro-Leu-Arg insertion creates a sharp kink in the polypeptide chain, which leads to 3D conformational changes in the extracellular domain and increases the distance between Cys38 and Cys97 (Fig. 5B, bottom). This model underscores the importance of the first Ig-like loop for CD19 transport through the ER and predicts that eliminating the disulfide bond between Cys38 and Cys97 would have the same effect on CD19 subcellular localization as skipping exon 2 or the 105R mutation.

**FIG 6:**
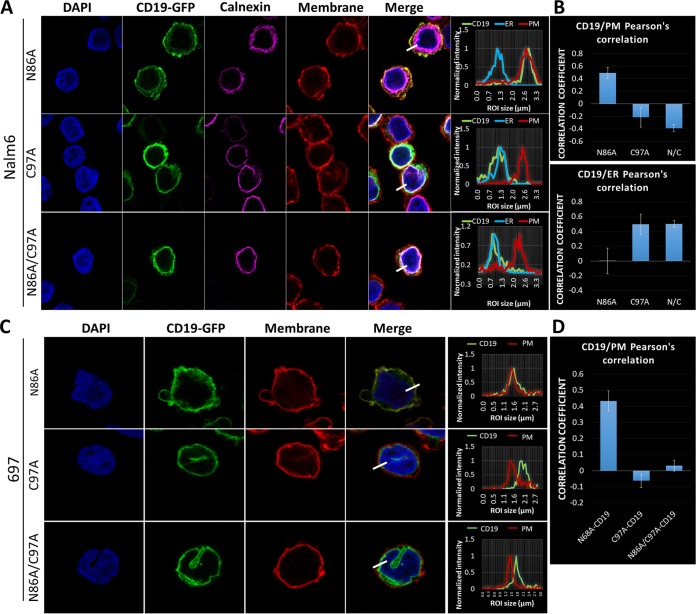
Disruption of the CD19 Cys38-Cys97 disulfide bond leads to endoplasmic reticulum retention. (A) Immunofluorescence confocal microscopy of the indicated CD19-GFP construct (green)-transduced Nalm6 cell lines. The plasma membrane was stained with wheat germ agglutinin-Alexa Fluor 647 (converted to red), the endoplasmic reticulum was stained with anticalnexin (Cell Signaling)/anti-rabbit antibody–Alexa Fluor 594 (converted to magenta), and nuclei were stained with DAPI (blue). (Right) Histogram localization analysis showing overlap of CD19-GFP, ER/calnexin, and plasma membrane channels. (B) Pearson's correlation colocalization analyses of green (CD19) and red (plasma membrane) channels or green (CD19) and ER/calnexin channels for the indicated Nalm6 cell lines. Three separate fields containing at least 100 cells were analyzed for each condition. The error bars indicate standard deviations. (C) Immunofluorescence confocal microscopy of the indicated CD19-GFP construct (green)-transduced 697 cells. The plasma membrane was stained with wheat germ agglutinin-Alexa Fluor 647 (converted to red), and nuclei were stained with DAPI (blue). (Right) Histogram localization analysis showing overlap of CD19-GFP (green) and the plasma membrane (red). (D) Pearson's correlation colocalization analyses of green (CD19) and red (plasma membrane) channels for the indicated 697 cell lines. Three separate fields containing at least 100 cells were analyzed for each condition.

To test this prediction, we generated both Cys97→Ala (C97A) and the double C97A/N86A CD19 mutants, both in the native conformation and fused to GFP (Fig. 5A). Swainsonine treatment of Nalm6 CD19-null cells expressing these constructs revealed that the C97A and C97A/N86A mutants lack sensitivity to swainsonine (Fig. 5C). The lack of gel shift was similar to that seen with the Δex2 CD19 mutant (Fig. 2C). This similarity was further confirmed when lysates from those cell lines were subjected to *in vitro* digestion with PNGase F (Fig. 5D). PNGase F experiments were reproduced in 697 cells (Fig. 5E) with similar results. Using live-cell flow cytometry for Nalm6 cells, we observed that both C97A and C97A/N86A mutants were invisible to the FMC63 antibody, although unlike Δex2 CD19, they retained the cognate amino acid sequence (Fig. 5F, top). The same results were obtained using 697 cells (Fig. 5F, bottom). Finally, confocal microscopy of cells expressing the GFP versions of C97A and C97A/N86A mutants showed that both had pronounced ER localization compared to the N86A mutant, which behaves similarly to FL-CD19 (Fig. 6A and B). These results were confirmed in 697 cells (Fig. 6C and D). All these findings fully support our hypothesis that preservation of the first Ig-like loop is critical for proper 3D folding of CD19 and its eventual trafficking to the plasma membrane.

### Endogenous CD19ex2 variants generated by genome editing are also retained in the endoplasmic reticulum.

**FIG 7:**
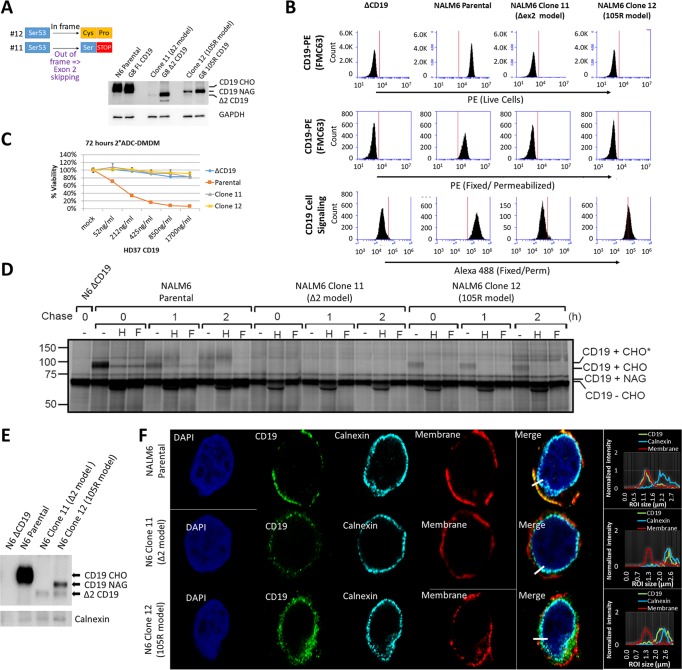
Endogenous CD19ex2 variants generated by genome editing are also retained in the endoplasmic reticulum. (A) Schematic of CRISPR/Cas9 mutant clones to model CD19-Δex2 (clone 11) or CD19-105R (clone 12). Shown is a Western blot with anti-CD19 or anti-GAPDH antibodies to compare protein expression between CRISPR/Cas9 and retroviral overexpression cell line models. (B) Flow cytometry of live or fixed and permeabilized Nalm6 parental and CRISPR/Cas9-modified cells expressing endogenous CD19 wild-type (parental), CD19-Δex2 (clone 11), and CD19-105R-like (clone 12) isoforms. (C) Killing assay with anti-CD19 antibody and anti-mouse DMDM 2-ADC using parental Nalm6 (CD19-WT), ΔCD19 (null), clone 11 (CD19-Δex2), or clone 12 (CD19-105R-like) cells. The error bars indicate standard deviations. (D) Nalm6-ΔCD19, parental, clone 11, and clone 12 cell lines were radiolabeled for 15 min, chased for 1 or 2 h, and immunoprecipitated using a monoclonal antibody against human CD19. Immunoprecipitates were treated with endo H or PNGase F before analysis on an SDS-PAGE gel. CHO, high-mannose-type glycans; CHO*, complex-type glycans; NAG, *N*-acetylglucosamine. (E) Immunoblotting with anti-CD19 or anticalnexin antibodies following immunoprecipitation with anti-CD19 antibody in the indicated cell lines. (F) Immunofluorescence confocal microscopy of parental Nalm6 (CD19-WT), clone 11 (CD19-Δex2), and clone 12 (CD19-105R-like) cells. The cells were stained with anti-CD19 (3G7; Origen)/anti-mouse antibody–Alexa Fluor 488 (green). The plasma membrane was stained with wheat germ agglutinin-Alexa Fluor 647 (converted to red), the endoplasmic reticulum was stained with anticalnexin (Cell Signaling)/anti-rabbit antibody–Alexa Fluor 594 (converted to cyan), and nuclei were stained with DAPI (blue). (Right) Histogram localization analysis showing overlap of color channels.

Retroviral expression can lead to gross overexpression and protein mislocalization. To validate our findings using endogenous CD19 variants, we used CRISPR/Cas9 genome editing with a single sgRNA that targets exon 2 to induce mutations that result in surface CD19 negativity. Nalm6 cultures were sorted for sCD19-negative cells, and their genomic DNA was analyzed to confirm the presence of mutations in exon 2. Pooled sCD19-negative cells were fluorescence-activated cell sorter (FACS) sorted into single-cell clones, expanded, and analyzed by deep targeted sequencing of exon 2 and Western blotting. We identified a clone that contains a homozygous out-of-frame C insertion, which can express only the Δex2 CD19 isoform via alternative splicing (Fig. 7A, number 11). We also identified another clone with a GCC insertion leading to the replacement of Ser53 with Cys-Pro (Fig. 7A, number 12). The insertion of Pro in clone 12 mimicked the mutation in 105R. Additionally, 3D i-Tasser analysis of clone 12 predicted that Cys53 could interact with Cys38, which would lead to disruption of the Cys38-Cys97 disulfide bond (data not shown). Clones 11 and 12 were confirmed by Western blotting to express, respectively, Δex2 and the immature CD19 variant (Fig. 7A). We also compared CD19 expression levels in CRISPR/Cas9-edited and retrovirus-transduced Nalm6 derivatives. As expected, the levels of CD19 expression in CRISPR/Cas9-edited clones were significantly lower than retroviral expression (Fig. 7A). Of note, CD19 levels in clones 11 and 12 were lower than CD19 levels in parental Nalm6 cells (Fig. 7A), making it a desirable model.

As hypothesized, the FMC63 antibody failed to recognize CD19 in clones 11 and 12 (whether live or fixed/permeabilized) in the same way it failed to detect retrovirus-overexpressed Δex2 CD19 (Fig. 7B, top and middle). However, using an antibody reactive with the cytoplasmic domain of CD19 (no. 3574; Cell Signaling), we readily detected its expression in fixed/permeabilized clone 12 (105R model) (Fig. 7B, bottom). On the other hand, Δex2 CD19 expressed by clone 11 was barely detectable, attesting to the inefficiency of alternative splicing and consistent with the Western blotting shown in Fig. 7A. To demonstrate that clones 11 and 12 were resistant to immunotherapy, we performed a 2-ADC killing assay, with parental Nalm6 cells as a positive control. As expected, clones 11 and 12 were as resistant to killing by this FMC-based immunotherapeutic as the Δex2 variant (Fig. 7C).

We then performed the pulse-chase experiment, and results obtained using clones 11 and 12 were similar to those seen with retrovirus-transduced cells (Fig. 7D). Specifically, we observed accumulation of complex glycans in the parental cells but not in the genome-edited clones, as evidenced by the absence of endo H resistance (Fig. 7D). Moreover, using coimmunoprecipitation (co-IP), we observed very weak binding of calnexin to the wild-type (WT) CD19 but increased binding to the mutant isoforms in clones 11 and 12 (Fig. 7E). Consistent with these co-IP data, confocal microscopy revealed colocalization of CD19ex2vs in clones 11 and 12 with the calnexin-marked endoplasmic reticulum (Fig. 7F). Collectively, these findings alleviated concerns about possible artifacts of retrovirus-based expression systems and allowed us to perform additional experiments for which expression of mutant CD19 was required.

### CD19ex2 variants preferentially interact with ER/major histocompatibility complex (MHC)-related proteins.

**FIG 8:**
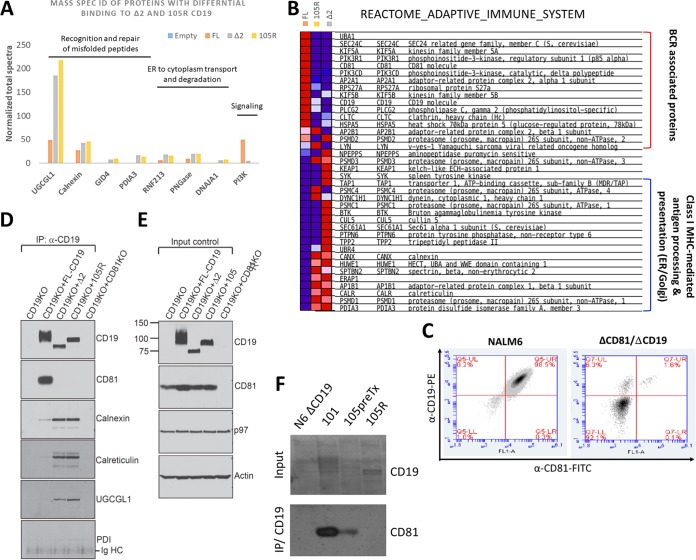
CD19ex2 variants preferentially interact with ER/MHC-related proteins. (A) Immunoprecipitates of CD19 from lysates of transduced Nalm6 cell lines were subjected to mass spectrometry. The bars represent normalized total spectra for the identified protein targets. (B) Heat map showing proteins from the Reactome Adaptive Immune System gene set that exhibited differential binding to CD19, CD19-105R, and CD19-Δex2, as determined by mass spectrometry. (C) Double-stained live-cell flow cytometry using anti-CD19–PE and anti-CD81–FITC antibodies on Nalm6 parental and Nalm6-ΔCD81 cell lines. (D) Immunoblotting of CD81, calnexin, calreticulin, UGCGL1, and PDI following immunoprecipitation with anti-CD19 antibody in lysates of the transduced cell lines. The CD19/CD81 double-KO Nalm6 cell line was used as a negative control. (E) Western blot for total levels of CD81, P97, and actin in lysates from the transduced cell lines. The CD19/CD81 double-KO Nalm6 cell line was used as a negative control. (F) Immunoblotting of CD81 following immunoprecipitation with anti-CD19 antibody in lysates from primary patient samples expressing CD19-WT (101 and 105) and CD19-105R immunotherapy-resistant isoforms.

We had previously shown that, upon stimulation of the B-cell receptor (BCR), Δex2 CD19 weakly interacts with phosphatidylinositol 3-kinase (PI3K) and Lyn, as measured by coimmunoprecipitation (11). To more completely understand the landscape of protein-protein interaction of CD19ex2vs, we performed liquid chromatography-tandem mass spectrometry (LC–MS-MS) analysis on CD19 coimmunoprecipitates. Prior to initiating these experiments, we had tested several CD19 antibodies and conditions for immunoprecipitation and chose the antibody that completely depleted the protein lysate of CD19. Nalm6 CD19-null cells expressing FL-CD19, Δex2 CD19, 105R, or the empty vector were stimulated for 8 min with anti-human IgM as described previously (30). Whole-protein lysates were incubated with anti-CD19 antibody, and pulldown fractions were analyzed by LC–MS-MS. The quality of the immunoprecipitation was also assessed by MS, ensuring that the CD19 protein itself received the top ranking. We confirmed that, compared to FL-CD19, Δex2 CD19 weakly interacts with PI3K (Fig. 8A). On the other hand, proteins involved in recognition and repair of misfolded proteins, ER-to-cytoplasm transport, and degradation were found to preferentially bind to the CD19ex2vs isoforms (Fig. 8A).

Subsequent gene set enrichment analysis (GSEA) (31) of FL-CD19 interactors revealed significant enrichment for proteins involved in adaptive immune response. For example, FL-CD19 was found to interact with many BCR-associated proteins (Fig. 8B). Interestingly, one of the top hits (right below PI3K) was the CD81 tetraspanin. Others have shown that CD81 and CD19 interact in the post-endoplasmic reticulum compartment and that this interaction is required for proper CD19 trafficking and surface presentation (32, 33). CD81 was notably missing from Δex2 and 105R interactors. Instead, these CD19ex2vs interacted with ER/Golgi apparatus-associated MHC class I (MHC-I)-mediated antigen-presenting proteins (Fig. 8B).

To confirm the LC–MS-MS findings, we first knocked out, using the CRISPR/Cas9 system, the CD81 gene from Nalm6 cells already negative for CD19 (Fig. 8C). We used this CD81/CD19 double-KO cell line as a negative control for coimmunoprecipitation experiments. As expected, CD81 was detectable in pulldowns from only FL-CD19 cells and not cells expressing any of the CD19ex2vs isoforms (Fig. 8D and E). In contrast, Δex2 and 105R mutants showed increased binding to ER-resident proteins, such as calnexin, calreticulin, UGCGL1, and PDI (Fig. 8D and E), fully consistent with the LC–MS-MS data. To assess the clinical relevance of our findings, we performed CD19 coimmunoprecipitations using protein lysates of primary patient samples: 101 WT CD19 pre-CART; 105 pretreatment (105preTx; previously referred to as CHOP105R1 [11]); and the matching relapse sample, 105R. As expected, only in samples that expressed WT CD19 and that were sensitive to the CART therapy (but not in exon 2-mutated CHOP105R) was there evidence of interaction between CD19 and CD81 (Fig. 8F).

## DISCUSSION

Understanding the molecular mechanisms behind antigen loss after targeted immunotherapy is critical to the design of alternative and complementary approaches. While in some epitope-negative relapses CD19 expression is extinguished at the level of dedifferentiation into myeloid lineages (9, 10), in our previous paper we described a novel mechanism of resistance to CD19-directed immunotherapy that involved alternative splicing of CD19 exon 2 (11, 34). This event inevitably leads to epitope loss, explaining why skipping of exon 2 would cause treatment failure. However, we also identified in-frame mutations in exon 2 that do not lead to the loss of amino acid sequences (e.g., the Pro-Lys-Arg insertion CHOP105R), yet CHOP105R was completely resistant to CART-19 immunotherapy.

Here, we demonstrate that CHOP105R and similar CD19 variants are invisible to the FMC63 antibody-based therapeutics (ADC and CAR T cells alike) for two related but distinct reasons. One is that conformational changes in the first Ig-like C2 loop of CD19 prevent recognition by the antibody even in permeabilized cells. In principle, this type of resistance could be addressed by designing an alternative immunotherapy directed at the second C2 loop in CD19. Surprisingly, the inclusion of the VSVg tag at the N terminus of the 105R CD19 variant did not result in cell killing by VSV-specific antibody-drug conjugates. Moreover, the tagged protein was not even expressed on the surfaces of transduced cells. Thus, the second reason for resistance to FMC63-based immunotherapies is the failure of CD19ex2vs to be transported to the plasma membrane. We anticipate the emergence of other immunotherapy-resistant cases that harbor mutations affecting the folding and trafficking of CD19 to the cell surface.

CD19 protein folding and transport are initiated as the nascent peptide enters the ER lumen through binding of a variety of resident chaperones. CD19 exon 2 variants appear to have lost their affinity for tetraspanin CD81, the key binding and transport partner of CD19, in whose absence CD19 is largely unable to undergo complex glycosylation and surface expression (31). Recently, one case of resistance to blinatumomab was attributed to CD81 loss, highlighting the importance of the CD19/CD81 interaction for responses to CD19-targeted immunotherapies (33). Interestingly, CD81 binds to the extracellular domain of CD19 (reviewed in reference 35), which remains intact in the 105R variant and partially intact in the Δex2 variant (36, 37), but apparently the preservation of the amino acid sequence is necessary but not sufficient for productive interactions between CD19ex2vs and CD81.

In parallel, resident chaperones, such as calnexin, PD1, and UGCGL1, acquire affinity for CD19ex2vs. All of them are part of the protein-folding quality control mechanism of the ER, and their preferential binding to CD19 ex2vs provides clear evidence that immunotherapy-resistant CD19 variants are misfolded. It is difficult to determine conclusively whether this misfolding is the cause or the consequence of CD81 unavailability. However, it has been previously observed that some full-length CD19 molecules are able to reach the plasma membrane even in CD81 KO cells, suggesting that misfolding of CD19ex2vs is the root of the problem (32).

Previous studies have shown that some mutations in surface receptors that lead to ER retention can be rescued by culturing the cells at a lower temperature or using so-called chemical chaperones. Here, we show that culturing B-ALL cells at 32°C modestly improves surface expression of some (but not all) of the CD19 variants, indicating that certain protein sequence alterations have a bigger impact on folding and cell surface expression than others. Unfortunately, even for the best responder (the CHOP105R variant), the increase in surface presentation induced by permissive temperature was insufficient to cause enough binding and/or internalization of the VSVg-specific ADC and cell killing. However, since CAR T cells do not rely on internalization and exhibit much higher antigen sensitivity, one could envision a scenario in which alternative non-FMC63-based CARs could be effective when combined with a rescue-from-the-ER approach. A high-throughput screening of chemical chaperones could be carried out to devise such an approach. Once identified, appropriate chemical chaperones could also be used to overcome low antigen density, which is emerging as another important mechanism of immune evasion (5, 34, 38). Furthermore, it may be possible to create T-cell receptor (TCR) mimic CARs against neoantigens to retain specificity and sensitivity of CAR-based immunotherapy (39).

Finally, GSEA of proteins bound to CD19ex2vs showed that misfolded variants are also bound to proteins that participate in antigen processing and presentation, such as calreticulin and TAP1 (40, 41). This finding suggests that these CD19 variants could be recognized by the MHC class I machinery. In fact, it has been recently shown that CD19 peptides can be found presented by MHC-I and -II in samples from primary human lymphomas (42). This opens the intriguing possibility of targeting sCD19-negative but misfolded CD19-positive tumors by T-cell receptors targeting MHC-presented CD19 peptides.

## MATERIALS AND METHODS

### Cell lines, cell culture, transfections, treatments, and infections.

Nalm6 and 697 cells were obtained from the Center for Childhood Cancer Research biorepository in 2009. B-lymphoid cell lines were cultured and maintained in RPMI 1640 medium supplemented with 10% fetal bovine serum (FBS), 2 mmol/liter l-glutamine, and penicillin-streptomycin at 37°C and 5% CO_2_. For the low-temperature experiments, cells were maintained at 32°C and 5% CO_2_ for at least 1 week. The U2OS and 293 cell lines were maintained in Dulbecco's modified Eagle's medium (DMEM) supplemented with 10% FBS. BCR ligation was performed by incubation of 20 × 10^6^ cells with 10 μg/ml of pre-BCR-specific anti-IgM antibody (IgM-5μ) (Jackson ImmunoResearch) or with isotype control goat anti-IgG (Southern Biotech; 0109-01) for the indicated times at room temperature. The cells were lysed in radioimmunoprecipitation assay (RIPA) buffer and loaded onto PAGE gels for immunoblotting analysis. pMX-CD19 retroviral constructs were cotransfected with Gag/Pol and VSVg constructs into 293 cells in RPMI medium. The medium was then filtered and used for transduction in Nalm6, 697, and U2OS cell lines. Cells were selected in 10 μg/ml blasticidin for 5 days.

### Cytotoxicity assays.

Cells grown at 37°C or 32°C were counted, and 5,000 cells/well were seeded in triplicate for each treatment and each cell line. Primary anti-VSVg mouse monoclonal antibody was added at 1:1,000 dilution, and the cells were incubated at 37°C for 10 min. A 2-ADC anti-mouse antibody–MMAF (Moradec; AM-202-AF) was then added at different final concentrations. After 96 h at 37°C, cell viability was measured using the Cell Titer-Glo luminescent cell viability assay (Promega; G7570) according to the manufacturer's protocol.

### CRISPR/Cas9 genome-editing system.

CD19- and CD81-CRISPR/Cas9-KO plasmids were obtained from Santa Cruz Biotechnologies (sc-400719 and sc-419571) and transfected into Nalm6 and 697 cell lines via electroporation using the AMAXA system program 0-006 and Reagent V (Lonza). Cells were stained with anti-CD19 or anti-CD81 PE-conjugated antibody (Beckman Coulter) 5 days after transfection. CD19-deficient (ΔCD19) cells were sorted and plated in 96-well clusters for single-cell clone selection and expansion. CD81-deficient (ΔCD81) cells were maintained as a pool. CD19 and CD81 knockdown was confirmed by flow cytometry and by Western blotting. DNA and RNA were extracted, and CD19 and CD81 genes were sequenced to analyze the mutations induced by the CRISPR/Cas9 system. To generate frameshift mutations into *CD19* exon 2, a single CRISPR/Cas9 exon 2-gRNA plasmid was transfected by electroporation into Nalm6 cells, as described above. Effective insertion of frameshift mutations in the expected targeted region was assessed by Sanger-sequencing screening of single-cell clones.

### Retroviral and lentiviral constructs.

pMX retroviral constructs expressing full-length and mutant *CD19* variants were generated by a 2-step PCR approach with primers that carry the desired mutation or a VSVg tag. WT CD19 cDNA from previously described (11) murine stem cell virus (MSCV)-internal ribosome entry site (IRES)-DsRedFP was used as a template. Digestion of pMX-IRES-CD19-GFP vector (10) with EcoRI/BlpI restriction enzymes, followed by ligation into pMX-IRES-blasticidin (RTV-016; Cell Biolabs) retroviral backbones, was performed. To generate *CD19* Δex2- and *CD19* Δex5-6-expressing vectors, the cDNA fragments were synthesized (Genewiz) and cloned into MSCV-CD19-IRES-DsRedFP via EcoRI/BglII or BglII/XhoI and later moved into pMX-IRES-BLAST via EcoRI/XhoI cloning. Retroviral and lentiviral particles were generated by transfection of GP293 cells with Lipofectamine-2000 (Invitrogen). Viral supernatants were harvested 24, 36, and 48 h after transfection and used to infect B-ALL cell lines in the presence of Polybrene (4 μg/ml). Where indicated, selection of infected cells was done with 10 μg/ml blasticidin over the course of 1 week or by cell sorting.

### Antibodies.

The following antibodies were used for protein detection: anti-CD19–PE (Beckman Coulter; IM1285U), anti-CD19 (Cell Signaling; 3574), anti-CD19 3G7 (Origen; TA506234), anti-CD19 HD37 (Thermo Fisher; MA1-34005), anti-VSVg–fluorescein isothiocyanate (FITC) (Abcam; ab3863), anti-VSVg (Abcam; ab50549), anti-CD81 (LifeSpan; LS-C359239), anti-CD81–FITC (Molecular Probes; A15753), anticalnexin (Cell Signaling; 2679), anticalreticulin (Cell Signaling; 12238), anti-UGCGL1 (NovoPro Laboratories; 116554), anti-PDI (Cell Signaling; 3501), anti-mouse antibody–Alexa Fluor 488 (Thermo Fisher; R37114), and anti-rabbit antibody–Alexa Fluor 594 (Thermo Fisher; A-21207).

### Immunofluorescence and colocalization studies.

The cells expressing CD19-GFP fusion variants were fixed for 10 min on ice with 4% paraformaldehyde, washed, and then stained with 5 μg/ml wheat germ agglutinin-Alexa Fluor 680 (Molecular Probes; W32465) according to the manufacturer's instructions. The cells were permeabilized with 0.2% saponin, 0.5% bovine serum albumin (BSA), 0.03 M sucrose in phosphate-buffered saline (PBS) for 10 min at room temperature. Primary calnexin antibody was incubated overnight at 4°C. Secondary Alexa Fluor 594–anti-rabbit antibody was incubated for 1 h at room temperature. The cells were the washed and mounted on precharged glass microscope slides with DAPI (4′,6-diamidino-2-phenylindole)-containing medium (Vectashield; H1200) and visualized under a Leica STED 3× superresolution confocal system (HC PL APO CS2 63×/1.40-numerical-aperture oil 63× objective). Images were acquired using 4,184-by-4,184 resolution with limited signal saturation. Colocalization was quantified by Pearson correlation coefficient. Six images for each CD19 construct containing 100 cells on average were analyzed with BioImageXD and Fiji Coloc2 plug-in software. The statistical Costes *P* value was 1 for this analysis (38).

### Flow cytometry.

Live or fixed and methanol-permeabilized cells were stained with PE-conjugated CD19 antibody (IM1285U; Beckman Coulter). The use of mouse HD37 CD19 or mouse CD81 was followed by anti-mouse Alexa Fluor 488-conjugated secondary antibody. Cells were analyzed in an AccuriC6 cytometer as previously described (3, 43).

### Western blotting, pulse-chase experiments, immunoprecipitation, and protein deglycosylation.

Whole-cell protein lysates were obtained in RIPA buffer. Protein concentrations were estimated by Bio-Rad colorimetric assay. Immunoblotting was performed by loading 20 μg of protein onto 10% PAGE gels. Signals were detected by enhanced chemiluminescence (Pierce). Immunoprecipitations were performed in whole-cell protein lysates from 15 million cells in 500 μl of nondenaturing buffer (150 mmol/liter NaCl, 50 mmol/liter Tris, pH 8, 1% NP-10, 0.25% sodium deoxycholate) and a 1:50 dilution of the corresponding specific antibodies. After overnight incubation at 4°C, 50 μl of protein A-agarose beads (Invitrogen) was added and incubated for 1 h at 4°C. The beads were washed 3 times with nondenaturing buffer, and proteins were eluted in Laemmli sample buffer, boiled, and loaded onto PAGE gels. For the pulse-chase experiments, cells were starved in methionine- and cysteine-free medium containing dialyzed fetal bovine serum for 1 h and pulse-labeled with 250 μCi/ml [^35^S]methionine and [^35^S]cysteine (Perkin-Elmer) for the indicated times. After labeling, the cells were incubated in the chase medium containing unlabeled methionine (2.5 mM) and cysteine (0.5 mM). At the end of each chase interval, the cells were lysed in RIPA buffer containing protease inhibitors. Precleared lysates were incubated with an anti-human CD19 antibody, together with protein G-Sepharose beads. For deglycosylation experiments, bead-bound CD19 was eluted and denatured in glycoprotein denaturing buffer (0.5% SDS, 40 mM dithiothreitol [DTT]) at 95°C for 10 min, followed by addition of sodium citrate (pH 5.5) to a final concentration of 50 mM, and incubated with endo H (New England BioLabs) at 37°C for 3 h. Alternatively, sodium phosphate (pH 7.5) and NP-40 were added to the denatured cell lysates to a final concentration of 50 mM and 1%, respectively, and the mixture was incubated with PNGase F (New England BioLabs) at 37°C for 3 h. Samples were boiled in SDS-PAGE sample buffer (62.5 mM Tris-HCl, pH 6.8, 2% SDS, 10% glycerol, 0.1% bromophenol blue) with β-mercaptoethanol, analyzed by SDS-PAGE, and visualized by autoradiography.

### Deglycosylation assay.

Protein lysates were obtained using a nondenaturing buffer (150 mmol/liter NaCl, 50 mmol/liter Tris, pH 8, 1% NP-10, 0.25% sodium deoxycholate) and treated with deglycosylation mixture (New England BioLabs; P6039S) following the manufacturer's instructions in the presence of protease and phosphatase inhibitors (Thermo Scientific; 78446). Control and deglycosylated lysates were loaded onto 10% PAGE gels for Western blot analysis.
